# Transcriptome datasets of β-Aminobutyric acid (BABA)-primed mono- and dicotyledonous plants, *Hordeum vulgare* and *Arabidopsis thaliana*

**DOI:** 10.1016/j.dib.2022.107983

**Published:** 2022-02-22

**Authors:** Géza Hegedűs, Ágnes Nagy, Kincső Decsi, Barbara Kutasy, Eszter Virág

**Affiliations:** a EduCoMat Ltd., Keszthely, Hungary; b Research Institute for Medicinal Plants and Herbs Ltd., Budakalász, Hungary; c Department of Plant Physiology and Plant Ecology, Hungarian University of Agriculture and Life Sciences, Georgikon Campus, Keszthely, Hungary; d Department of Molecular Biotechnology and Microbiology, Faculty of Science and Technology, Institute of Biotechnology, University of Debrecen, Hungary

**Keywords:** β-Aminobutyric acid, *Arabidopsis thaliana*, *Hordeum vulgare*, Transcriptome, Illumina sequencing

## Abstract

The non-protein amino acid β-Aminobutyric acid (BABA) may trigger the immune responses of plants to various biotic and abiotic stresses leading to a long-term resistance (primed state). We present RNA-seq datasets of BABA - primed mono- and dicotyledonous plants, such as *Arabidopsis thaliana* and *Hordeum vulgare*. Illumina NextSeq550 sequencing were carried out after 72 h of BABA exposure. 87 bp long sequence reads were preprocessed of treated and control samples and deposited in the NCBI SRA database. Transcriptome datasets were de novo assembled of each species and deposited in the NCBI TSA database. These SRA and TSA depositions are under the Bioproject accession: PRJNA791573. Pairwise differential expression with enrichment analyses were performed and the most specific DEGs were determined and annotated in both plants.

## Specifications Table

**Table:** 

Subject	Plant Science: Plant Physiology
Specific subject area	Genome-wide transcription profiling as a response to BABA exposure were performed and compared between monocotyledonous and dicotyledonous plants, *A. thaliana* and *H. vulgare*.
Type of data	Table Database record Figure
How the data were acquired	Exogenous treatments of BABA were performed in phytotron experiments. The cultivation and exogenous (soil drench) BABA treatment of plants was accomplished in plant growth chambers. Approximately 30 mg of plant tissues (leaves) were used to prepare NGS libraries of BABA treated and control samples of *H. vulgare* and *A. thaliana*. NextSeq550 sequencing were performed resulting in 20M 87 bp long reads in each sample, approximately. Reads were pre-processed and assembled. Transcriptome datasets were reconstructed using combined read sets per species and separated per all samples. Genome wide expression profile in response for BABA treatment were determined by pairwise differential expression with gene set enrichment analysis. Differentially expressed genes (DEGs) were annotated and GO terms were identified.
Data format	Raw Analysed Filtered
Description of data collection	Plant growth chamber type was MLR352HPA -115V NEMA 5-20, 220V / 60Hz – Panasonic. Treatment conditions were as follows: temperature during the 1. day and night were 25°C. The temperature during the 2-16. days and nights were 25°C and 15°C. Duration of the day was 12 h, 04-4 p.m. Treatments were as follows: BABA (MW: 103.121 g/mol), final concentration in soil was 25 μM (soil drench). BABA treatment was performed at the day 14 and sample collection was performed at the day 17.
Data source location	• EduCoMat Ltd • Keszthely • Hungary
Data accessibility	The bio project and raw reads are available in National Center for Biotechnology Information database under the accessions: Repository name: Characterization of BABA-primed state of monocotyledonous and dicotyledonous plants Raw sequence reads Data identification number: PRJNA791573 Direct link to datasets: https://www.ncbi.nlm.nih.gov/bioproject/PRJNA791573 Repository name: RNA-seq of Arabidopsis thaliana: control leaf Data identification number: SRR17320108 Direct link to datasets: https://www.ncbi.nlm.nih.gov/sra/?term=SRR17320108 Repository name: RNA-seq of Arabidopsis thaliana: BABA treated leaf Data identification number: SRR17320107 Direct link to datasets: https://www.ncbi.nlm.nih.gov/sra/?term=SRR17320107 Repository name: RNA-seq of *Hordeum vulgare*: control leaf Data identification number: SRR17320106 Direct link to datasets: https://www.ncbi.nlm.nih.gov/sra/?term=SRR17320106 Repository name: RNA-seq of *Hordeum vulgare*: BABA treated leaf Data identification number: SRR17320105 Direct link to datasets: https://www.ncbi.nlm.nih.gov/sra/?term=SRR17320105

## Value of the Data

• These data contribute to the knowledge of genetic background of pathogen-free immune priming of plants. Immune response stimulation may differ in mono- and dicotyledonous plants, the presented data represent information for both classes.
• There is an emerging role of sustainable plant protection which may benefit from these data. The effect of priming-active elicitor, BABA on protection and signalling pathways has already been demonstrated, so its potential use in the agriculture is well-founded and highlighted.
• Illumina GEx sequencing represents the whole transcriptomic gene expression profiling. There is a rare data on the direct exposure and comparison of BABA for taxonomically distant monocotyledonous and dicotyledonous plants in an experiment. Our datasets may base the understanding the underlying differences of plant physiological processes helping fundamental and applied research as well.

## 1 Data Description

**Fig. 1:**
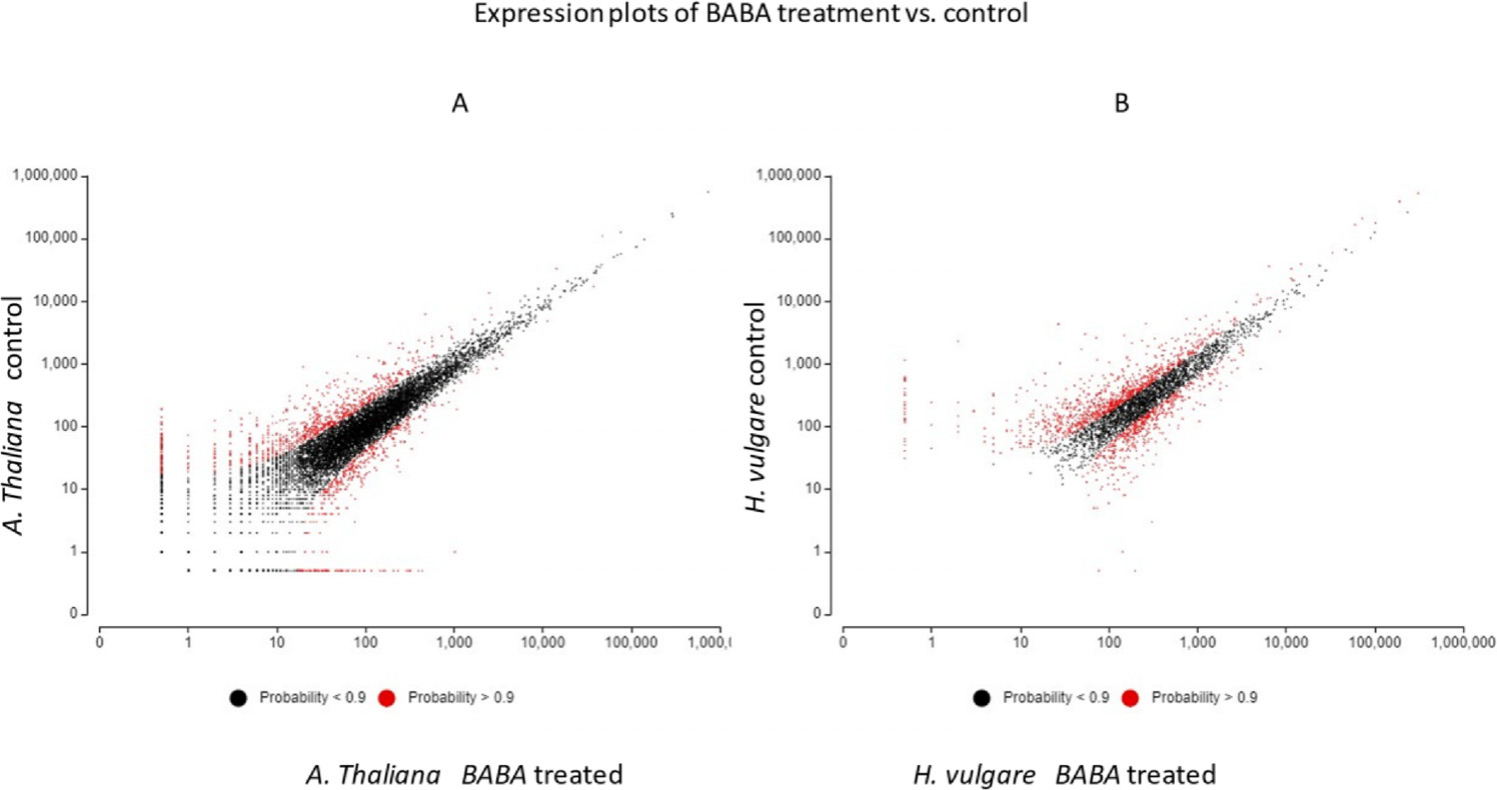
Expression plot of DEGs of *A. thaliana* and *H. vulgare*. The scatter plots show the average expression values of each condition. Differentially expressed features considering the probability threshold (0.9) are highlighted in red.

**Fig. 2:**
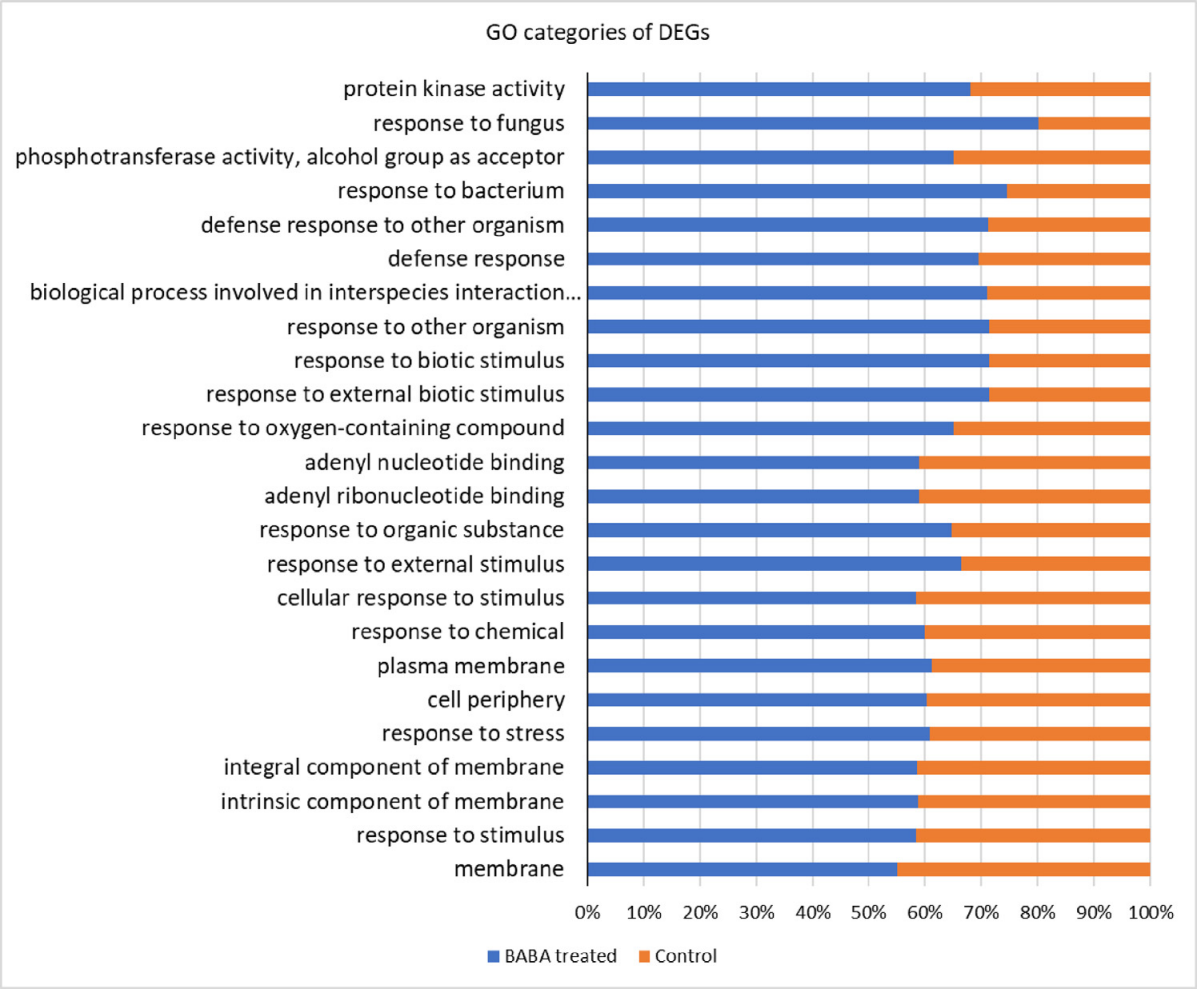
Most specific up-regulated GO categories as response to BABA treatment in *A. thaliana*.

**Fig. 3:**
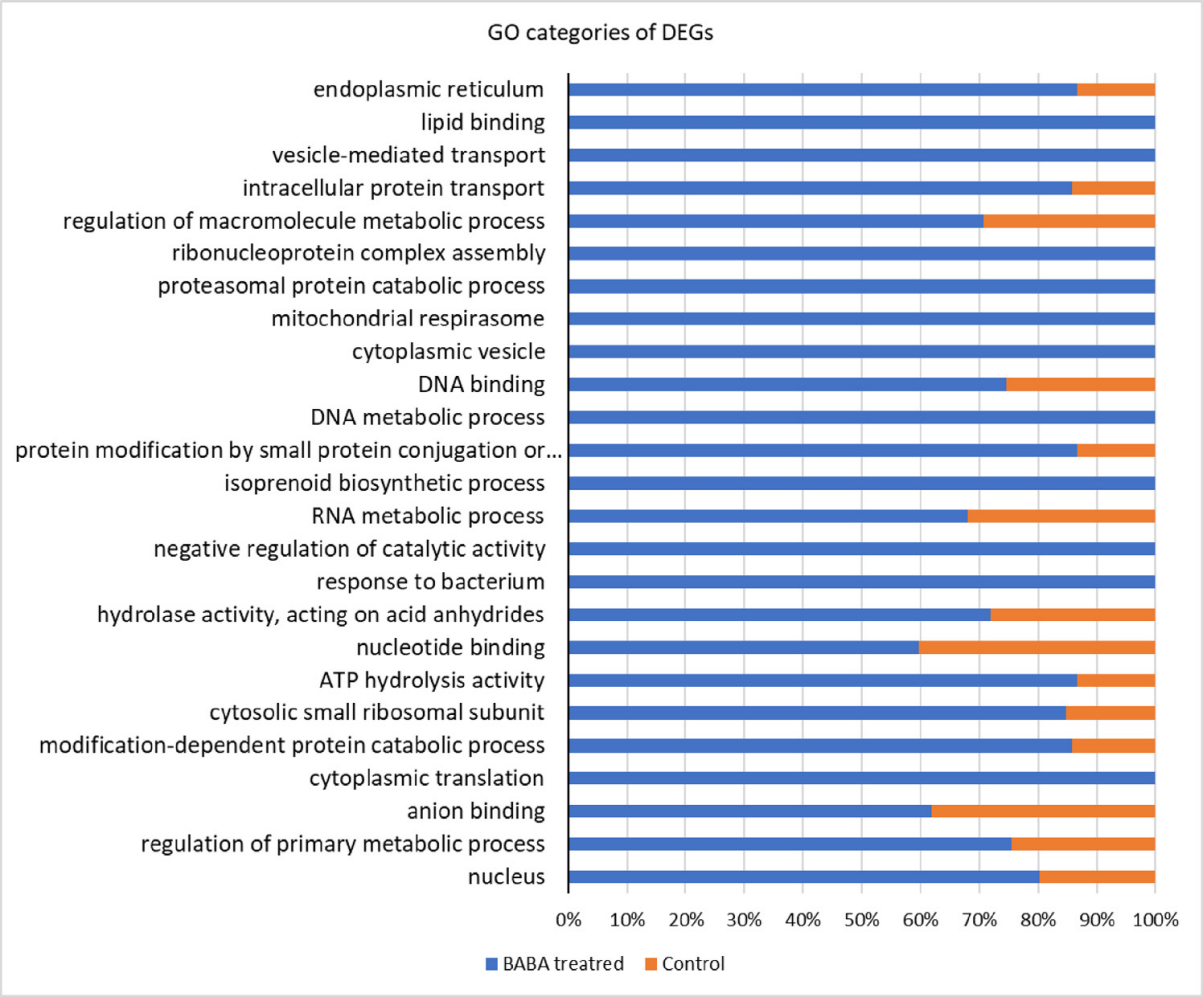
Most specific up-regulated GO categories as response to BABA treatment in *H. vulgare*.

Plant defence mechanisms against pathogens can be triggered by various inducers, of which BABA has been shown to be highly effective against viruses, bacteria, fungi, oomycetes, nematodes arthropods and abiotic stresses in several studies [1], [2], [3], [4], [5]. Shallow RNA-sequencing for gene expression profiling upon BABA exposure of the two classes of Angiosperms - dicotyledons and monocotyledons - plants, such as *A. thaliana* and *H. vulgare* are presented here. Sequence raw reads of BABA treated and control (non-treated) samples of *A. thaliana* and *H. vulgare* are deposited in the NCBI Sequence Read Archive (SRA) database under the Bioproject PRJNA791573 with accession numbers SRR17320108, SRR17320107, SRR17320106, SRR17320105. Using these SRA datasets de novo assemblies were performed for both species and Transcriptome Shotgun Assemblies (TSA) has been deposited at DBJ/EMBL/GenBank under the accessions GJRJ00000000, GJRK00000000. The versions described in this paper are the first versions, GJRJ01000000, GJRK01000000. Differentially expressed genes (DEGs) were determined aligning the SRA reads to the TSA datasets. These abundances are indicated in the CountTables. The CountTable of *A. thaliana* and *H. vulgare* BABA treated and control samples are presented in Supplemental Table 1 and Supplementary Table 2. The average expression values of DEGs are visualized in Fig. 1. Functional annotation data of DEGs of *A. thaliana* and *H. vulgare* are detailed in Supplemental Tables 3 and 4 of which the filtered top 25 gene ontology (GO) categories are visualized in Figs. 2 and 3.

## 2 Experimental Design, Materials and Methods

### 2.1 Plant materials

*H. vulgare* cv. Nure and *A. thaliana* cv. Columbia plants were cultured in phytotrons. BABA pretreatment was performed on 14-day-old plants. Fresh leaves of 17-day old plant samples were collected. Samples were stored in DNA/RNA Shield (Zymo research) at -25°C until sequencing.

### 2.2 NGS Library preparation and sequencing

For Gene Expression Profiling (GEx) library construction, QuantSeq 3‘mRNA-Seq Library Prep Kit FWD for Illumina (Lexogen GmbH, Wien, 510 Austria) was applied according to the manufacturer's protocol. Libraries were diluted to 1.8 pM for 1 × 87 bp single-end sequencing with 75-cycle High Output v2 Kit on the NextSeq 550 Sequencing System (Illumina, San Diego, CA, USA) according to the manufacturer's protocol. QuantSeq FWD allows to exactly pinpoint the 3’ end of poly(A) RNA and therefore obtain accurate information about the 3’ UTR. Using this sequencing fragments of coding sequences are 260–300 bp long on average.

### 2.3 Pre-processing and assembly

Reads were pre-processed using Trimmomatic software [6]. During this step adapters and contamination sequences were removed, low quality bases, short and low-quality reads were filtered out. Full-length transcriptome assembly of cleaned and combined read sets (*A. thaliana* combined, *H. vulgare* combined) from shallow RNA-Seq data were performed by using Trinity and Bowtie2 [7,8]. Output statistics of transcriptomes are summarized in Table 1.

**Table 1:** Transcriptome RNA-Seq de novo assembly results.

	*A. thaliana* combined	*H. vulgare* combined
Total transcripts	2115	2398
Total genes	1898	2248
Percent GC	37.55	44.33
Total assembled bases	618,514	789,082
N50	302	339

### 2.4 Gene level quantification

To estimate gene expression from RNA-sequencing CountTable was created. To count how many reads map to each feature of interest (genes) each sample reads were aligned to the combined transcriptomes (Table 1). Count Table creation was performed with OmixBox.BioBam (https://www.biobam.com/omicsbox/) using the HTseq package [9] and Bowtie2 [8]. Based on the data of CountTable DEGs were determined as response to BABA treatment with both species (Supplemental Tables 1 and 2).

### 2.5 Pairwise differential expression analysis

Numerical analysis of DEGs in a pairwise comparison of two different experimental conditions was carried out using OmixBox.BioBam. The used application is based on the RSEM and edgeR program implementing quantitative statistical methods to evaluate the significance of individual genes between two experimental conditions [10,11]. TMM (Weighted trimmed mean of M-values) normalization method was performed. Distribution of DEGs is shown in the Fig. 1.

### 2.6 Functional annotation

Annotation of pairwise differential expression with most specific DEGs were determined by Fisher's Exact Test. Overexpressed GO categories (biological process, molecular function and cellular component) as the response to BABA treatment are indicated in bar chart of both plants (Figs. 2 and 3). Gene set enrichment analysis with GO names, GO categories and statistics are detailed in Supplemental Table 3 and 4.

## CRediT authorship contribution statement

**Géza Hegedűs:** Software, Investigation. **Ágnes Nagy:** Conceptualization, Investigation. **Kincső Decsi:** Validation. **Barbara Kutasy:** Validation. **Eszter Virág:** Writing – original draft, Visualization, Supervision.

## Declaration of Competing Interest

The authors declare that they have no known competing financial interests or personal relationships that could have appeared to influence the work reported in this paper.

## Appendix Supplementary materials

Image, application 1

Image, application 2

Image, application 3

Image, application 4

## References

[1] Jakab G., Cottier V., Toquin V., Rigoli G., Zimmerli L., Métraux J.P., Mauch-Mani B. (2001). β-aminobutyric acid-induced resistance in plants. Eur. J. Plant Pathol..

[2] Wang K., Li C., Lei C., Zou Y., Li Y., Zheng Y., Fang Y. (2021). Dual function of VvWRKY18 transcription factor in the β-aminobutyric acid-activated priming defense in grapes. Physiol. Plant..

[3] Prieto J.D., Alomar O., Agustí N., Battaglia D., Fanti P., Trotta V., Castañé C. (2021). Does the plant defense priming compound β-aminobutyric acid affect the performance of Macrolophus pygmaeus when used to control Bemisia tabaci in tomato?. Phytoparasitica.

[4] Lee J.H., Anderson A.J., Kim Y.C. (2021). Induced resistance against a bacterial disease by orysastrobin, a chemical fungicide. Eur. J. Plant Pathol..

[5] Choudhary A., Kumar A., Kaur H., Balamurugan A., Padhy A.K., Mehta S. (2021). Plant Performance Under Environmental Stress.

[6] Bolger A.M., Lohse M., Usadel B. (2014). Trimmomatic: a flexible trimmer for Illumina sequence data. Bioinformatics.

[7] Grabherr M.G., Haas B.J., Yassour M., Levin J.Z., Thompson D.A., Amit I., Adiconis X., Fan L., Raychowdhury R., Zeng Q. (2011). Trinity: reconstructing a full-length transcriptome without a genome from RNA-Seq data. Nat. Biotechnol..

[8] Langmead B., Salzberg S.L. (2012). Fast gapped-read alignment with Bowtie 2. Nat. Methods.

[9] Anders S., Pyl P.T., Huber W. (2015). HTSeq-a python framework to work with high-throughput sequencing data. Bioinformatics.

[10] Robinson M.D., McCarthy D.J., Smyth G.K. (2010). edgeR: a Bioconductor package for differential expression analysis of digital gene expression data. Bioinformatics.

[11] Li B., Dewey C.N. (2011). RSEM: accurate transcript quantification from RNA-Seq data with or without a reference genome. BMC Bioinform..

